# Patients' experiences of behaviour change interventions delivered by general practitioners during routine consultations: A nationally representative survey

**DOI:** 10.1111/hex.13221

**Published:** 2021-03-04

**Authors:** Chris Keyworth, Tracy Epton, Joanna Goldthorpe, Rachel Calam, Christopher J. Armitage

**Affiliations:** ^1^ Manchester Centre for Health Psychology Divisional of Psychology and Mental Health School of Health Sciences Faculty of Biology, Medicine and Health The University of Manchester Manchester UK; ^2^ NIHR Greater Manchester Patient Safety Translational Research Centre The University of Manchester Manchester Academic Health Science Centre Manchester UK; ^3^ Manchester University NHS Foundation Trust Manchester Academic Health Science Centre Manchester UK

**Keywords:** behaviour change, general practitioners, health policy, health‐care professionals, patient education, prevention, primary care

## Abstract

**Background:**

Consistent with the ‘Making Every Contact Count’ UK public health policy, general practitioners (GPs) are expected to provide patients with behaviour change interventions opportunistically. However, there is a belief widely held among GPs that patients neither want or need such interventions. We aimed to understand the following: (a) the characteristics of people attending GP appointments, (b) patients' needs for health behaviour change, (c) perceptions of appropriateness and helpfulness of interventions, and (d) factors associated with recall of receipt of interventions.

**Methods:**

Cross‐sectional nationally representative online survey of UK adults who had attended GP clinics in the preceding four weeks (n = 3028). Data were analysed using descriptive statistics and binary logistic regression.

**Results:**

94.5% (n = 2862) of patients breached at least one health behaviour guideline, and 55.1% reported never having had a conversation with their GP about health behaviours. The majority of patients perceived intervention as appropriate (range 84.2%‐87.4% across behaviours) and helpful (range 82.8%‐85.9% across behaviours). Being male (OR = 1.412, 95% CI 1.217, 1.639), having a long‐term condition (OR = 1.514, 95% CI 1.287, 1.782) and a higher number of repeat GP visits (OR = 1.016, 95% CI 1.010, 1.023) were among factors associated with recall of receipt of interventions.

**Conclusions:**

Patients perceived behaviour change intervention during routine GP consultations as appropriate and helpful, yet there are variations in the likelihood of receiving interventions according to sociodemographic factors. GPs could adopt a more proactive approach to behaviour change in patient consultations with the broad approval of patients.

**Patient or public contribution:**

The questionnaire was piloted among a convenience sample prior to distribution.

## 1 BACKGROUND

Non‐communicable diseases (NCDs; cardiovascular disease, cancers, chronic respiratory diseases and diabetes) kill 41 million people each year.^1^ Health behaviours such as smoking, excessive alcohol intake, physical inactivity and unhealthy diet are all key risk factors of NCDs and place a heavy financial burden on the National Health Service (NHS).^2^ The recent Health Survey for England showed that 87% of adults breached national guidance on health behaviour.^3^

General practitioners (GPs) are often the first point of contact for patients consulting with the health service and are consequently well placed to offer brief interventions to support patients in improving their health behaviour. Public health strategies in the UK emphasize the need for preventive strategies as central to every patient contact.^4^ However, research to date provides a mixed picture of the extent to which GPs are willing to deliver behaviour change interventions. Some research suggests that GPs are dissatisfied by delivering behaviour change interventions such as smoking cessation.^5^ GPs may also feel they are actively discouraged from delivering interventions, in favour of addressing and managing the primary medical complaint.^6^ In contrast, other research suggests that GPs consider interventions for smoking cessation^5^ and weight management^7^ to be important clinical tasks, and part of their responsibility in routine clinical practice,^8^ even within the constraints of a time‐limited consultation. Consistent with this mixed pattern of findings, there is evidence that some GPs do deliver behaviour change interventions during routine practice, although the proportion of patients receiving such interventions is low. A recent national survey of health‐care professionals showed that GPs perceived that, of all the patients they saw in a typical week, 44% would benefit from a behaviour change intervention. However, GPs reported delivering interventions to just 34% of those patients who they perceived would benefit.^9^ Moreover, it is currently unknown how many patients attending GP appointments perceive they would benefit from a behaviour change intervention meaning that GPs currently make decisions based on their perceptions of patient need.

Findings from systematic reviews show that some GPs believe that: (a) patients may not want or need behaviour change interventions, and patients would perceive this information as inappropriate^10^, ^11^; and (b) patients may lack the motivation and desire for behaviour change and are unwilling and lack the motivation to modify their health behaviour.^12^ Consequently, GPs appear to make conscious decisions about whether or not to deliver interventions to patients, which may be influenced by biases towards certain types of patients based on GPs' perceptions of patient risk, and how receptive GPs believe that patients will be to conversations about health behaviours.^5^, ^8^, ^13^ It would therefore be valuable to examine the factors associated with recall of receiving health behaviour change interventions.

Despite the conflicting evidence in relation to GPs' perceptions about the value of behaviour change activities during routine practice, more research is needed focussed on patient perspectives. A recent qualitative study suggests behaviour change interventions are perceived by patients as appropriate and helpful during routine GP consultations, particularly where it would benefit long‐term condition management.^14^ Some research has examined patients' willingness to receive opportunistic behaviour change interventions within specific health contexts (eg behaviour change advice during cancer screening appointments^15^), but have not considered consultations for conditions that may have little to do (at least from the perspective of the patient) with the target health behaviour. It is important to understand the views of patients' perceptions of the delivery of behaviour change interventions during GP appointments for two reasons. First, it is currently unclear how many patients attending GP appointments breach national health behaviour guidelines. Filling this gap in knowledge would enable a clearer understanding of (a) patients' perceived need for behaviour change interventions as opposed to health‐care professionals' perceptions of patient need, and (b) the characteristics of people attending GP appointments who may need behaviour change interventions during GP appointments, and whether such characteristics are associated with participants' recall of receiving interventions. Second, it would be valuable to examine whether patients' experiences and perceptions of receiving behaviour change interventions are consistent with a view that is common among health‐care professionals, namely, that patients do not need or want behaviour change interventions.

The aims of the present study were to understand the following: (a) the characteristics of patients attending GP appointments, (b) patient need for behaviour change interventions, (c) acceptability of behaviour change intervention during routine GP consultations (perceived appropriateness and helpfulness) and (d) the factors associated with recall of receipt of receiving behaviour change interventions.

## 2 METHODS

### 2.1 Design and procedure

A cross‐sectional survey design was used. Patients who had visited their general practitioner (GP) at least once in the previous four weeks for a routine appointment (characterized as a pre‐scheduled appointment with a GP for any reason) were recruited via a survey panel (YouGov) in 2018. Members of YouGov's online panel are incentivized to take part in surveys, whereby respondents accumulate points for completing surveys, which can be exchanged for entry into prize draws or cash payment. Prior to distribution, to ensure there were no layout, formatting or technical issues, the questionnaire was piloted among a convenience sample (n = 430) consisting of YouGov's existing panel members. Ethical approval was obtained from a university ethics committee (ref 2018‐3662‐5925), and informed consent was obtained from participants at the beginning of the questionnaire. The data were collected and collated by YouGov and sent securely to the research team for analysis.

### 2.2 Participants

Purposive sampling was used to recruit participants to take part in an online questionnaire. Initially, a sample designed to be representative of adults resident in the United Kingdom was asked a screening question: ‘have you consulted a general medical practitioner (ie a GP doctor) within the last four weeks?’. Response options were as follows: ‘yes, in a local GP clinic’, ‘yes, in a hospital or accident and emergency department’, ‘yes, other’, ‘no’, or ‘don't know’. The final sampling frame was based on the respondents answering ‘yes, in a local GP clinic’.

### 2.3 Measures

The questionnaire, as part of a larger programme of research examining perceptions of health and well‐being, collected demographic information including age, gender, ethnicity and socioeconomic status. Patients' current health and any existing long‐term conditions that may be relevant to their visit were also examined. The EQ‐VAS^16^ was used for participants to provide a global assessment of their current health. Participants are asked to report their current health, using a visual analogue scale, on a scale of 0 (worst imaginable health), to 100 (best imaginable health). Participants were also asked: ‘Was the purpose of your last doctor (GP) visit to discuss a chronic condition (generally, these progress slowly, over an extended period of time, for example arthritis, high blood pressure, osteoporosis)’. For those indicating ‘yes’, participants were asked to complete the Functional Comorbidity Index^17^ which contains a list of 18 long‐term health conditions. An ‘other’ option was also provided for (a) other chronic conditions not covered in the Functional Comorbidity Index, (b) acute conditions (defined as ‘developing suddenly and often last a short time, for example a broken bone, common cold, flu’) or (c) any other reason not mentioned (defined as ‘including one‐off visits such as for a flu jab or health certificate’).

Participants were asked questions about their health behaviours, including self‐reported smoking status, alcohol intake, diet and physical activity. Smoking status was assessed using the question ‘Do you smoke cigarettes at all nowadays?’, with the responses ‘yes’ and ‘no’.^18^ Body mass index (BMI) was calculated from participants' self‐reported height and weight.

Alcohol, diet, physical activity and BMI were assessed using questions that allowed comparisons between participants' current health behaviours with government guidelines. Alcohol intake was assessed using the question ‘if you drink at all, how many units of alcohol do you have on a typical day when you are drinking?’ with responses on a 0‐30 units scale. Diet was assessed using the question ‘government guidelines suggest that a balanced diet includes at least five portions of a variety of fruit and vegetables every day. Additionally, adults' recommended daily intake of calories is 2000 for women, and 2500 for men. In an average week, how close are you to achieving this?’ with response options ‘you achieve this every week’, ‘you're almost there, but not quite’, ‘you do around half of what's recommended’ and ‘you're a long way off doing what's recommended.’ Physical activity was assessed using the question ‘Government guidelines suggest that adults should aim to be active daily. Over a week, adults should do at least 150 minutes (2 and a half hours) of moderate intensity activity, for example 30 minutes on at least 5 days a week. Alternatively, 75 minutes of vigorous intensity activity, spread across the week. Examples of moderate intensity physical activity include brisk walking and cycling; vigorous intensity physical activity includes running, and sports such as swimming. In an average week, how close are you to achieving this?’ with response options ‘you achieve this every week’, ‘you're almost there, but not quite’, ‘you do around half of what's recommended’ and ‘you're a long way off doing what's recommended’. The data were coded to allow comparisons between patients breaching national guidance on health behaviour, and those patients meeting the national guidelines.

To examine patients' experiences of the receipt of health behaviour interventions during routine GP appointments, participants were asked to estimate (a) the total number of times their GP asked about their health behaviour (approximately, in the preceding 12 months), (b) the total number of times each health behaviour was discussed (approximately, in the preceding 12 months), and (c) whether a referral was made in relation to further support for health behaviours.

To examine perceptions of the receipt of health behaviour interventions during routine consultations, for smoking, diet, physical activity, alcohol intake and weight management, participants were asked to rate the following: (a) perceived helpfulness of receiving a behaviour change intervention, (b) perceived appropriateness of receiving a behaviour change intervention, (c) perceived expectation of receiving a behaviour change intervention and (d) perceived concern as a result of receiving a behaviour change intervention. Perceived helpfulness and appropriateness were based on questions previously used in the literature.^19^ In relation to perceived helpfulness, participants were asked the question ‘In general, how helpful or not do you think it is for doctors (GPs) to ask people about the following?’ answered using the response options ‘very helpful’, ‘helpful’, ‘neither helpful nor unhelpful’, ‘not helpful’ and ‘not at all helpful’ in relation to each of smoking, diet, physical activity, alcohol intake and weight management. In relation to perceived appropriateness, participants were asked the question ‘In general, how appropriate or not do you think it is for doctors (GPs) to ask people about the following?’ answered using the response options ‘very appropriate’, ‘appropriate’, ‘neither appropriate nor inappropriate’, ‘not appropriate’ and ‘not at all appropriate’ in relation to each health behaviour. Perceived concern was answered using a 0 (not at all concerned) to 10 (very concerned) rating scale, adapted from Klein.^20^ Perceived expectation was answered using a 0 (never expect) to 10 (always expect) rating scale, developed by the research team in the absence of a standardized item measuring perceived expectation.

### 2.4 Analyses

Descriptive statistics were used to quantify (a) the characteristics of patients attending a GP appointment, (b) how many patients would benefit from behaviour change advice according to patients' self‐reported health behaviours, (c) ratings of experiences of discussing health behaviours during routine GP appointments and (d) ratings of acceptability (perceived helpfulness, appropriateness, expectation and concern) in relation to receiving behaviour change advice. Results are presented according to patients breaching versus meeting national health behaviour guidelines. Chi‐square was used to compare our sample of patients who attended a GP clinic with the general population. Binary logistic regression was used to examine the factors associated with recall of receipt of receiving behaviour change interventions, according to sociodemographic variables, peoples' current health status, health behaviours and perceptions of acceptability of behaviour change advice. The main outcome (discussion of behaviour change advice at last GP visit) was recorded as a binary outcome (ie yes [1] or no [0]).

## 3 RESULTS

### 3.1 Overview of sample

Of all the people who had recently attended a GP appointment (N = 3028), most were women (53.5%) and White British (84.2%), with a mean age of 50 years (SD = 16.78). Participants reported an average of nine GP appointments in the preceding 12 months (range 1‐100 visits). Table 1 shows an overview of our sample compared to national data. People who had recently attended a GP appointment closely resembled the general population in terms of gender, ethnicity and social grade. However, our sample of people attending a GP appointment in the preceding four weeks contained a lower proportion of adults aged 18‐34, a higher proportion of adults aged 55‐64 and a higher proportion of people in higher managerial, administrative and professional occupations, compared to national data. Our sample closely resembled national figures for BMI, smoking status, alcohol consumption and diet. However, a higher proportion of people who had recently attended a GP appointment did not meet physical activity guidelines (n = 2101; 69.4%) compared to national data (42%).

**TABLE 1:** Sample characteristics

Variable	n	(%)	Mean	(SD)	General population data (%)^a^, ^b^, ^c^, ^d^	*χ* ^2^ for difference between sample and population
Gender (%)				na		
Male	1408	(46.5)			(49.3)	0.16 (*P* = .69)
Female	1620	(53.5)			(50.7)	0.16 (*P* = .69)
Total	3028					
Age, years			49.77	(16.78)		
18‐25	276	(9.1)			(12.8)	37.05 (*P* < .001)
26‐34	414	(13.7)			(15.5)	7.47 (*P* < .05)
35‐44	479	(15.9)			(16.0)	0.02 (*P* = .88)
45‐54	547	(18.1)			(17.7)	0.33 (*P* = .56)
55‐64	574	(19.0)			(14.9)	40.05 (*P* < .001)
>64	731	(24.1)			(23.0)	2.06 (*P* = .15)
Social grade^h^						
A	467	(15.4)			(4)	31.51 (*P* < .001)
B	668	(22.1)			(23)	0.06 (*P* = .81)
C1	676	(22.3)			(28)	1.70 (*P* = .18)
C2	470	(15.5)			(20)	1.00 (*P* = .32)
D	293	(9.7)			(15)	1.96 (*P* = .16)
E	454	(15.0)			(10)	2.78 (*P* = .10)
Total	3028					
Ethnicity						
White British	2549	(84.2)			(87)	0.80 (*P* = .37)
Other/prefer not to say	479	(15.8)			(13)	0.80 (*P* = .37)
Total	3028					
GP visits in the last 12 months			9.27	(12.80)		
Body Mass Index (BMI)^e^			27.50	(6.17)		
Underweight	113	(4.0)			(2)	2.04 (*P* = .15)
Healthy Weight	958	(33.8)			(35)	0.04 (*P* = .83)
Overweight	943	(33.2)			(37)	0.69 (*P* = .41)
Obese	700	(24.7)			(24)	0.06 (*P* = .81)
Severely Obese	124	(4.4)			(3)	0.34 (*P* = .56)
Did not answer	190					
Total	3028					
BMI risk						
High risk ≥ 25	1767	(64.8)			(64)	0.04 (*P* = .83)
Low risk < 25	1261	(35.2)			(36)	0.04 (*P* = .83)
Current smoker						
Yes	476	(15.7)			(18.5)	0.59 (*P* = .24)
No	2552	(84.3)			(81.5)	0.59 (*P* = .24)
Alcohol consumption^f^						
Excessive consumption	566	(22.7)			(28.4)	1.24 (*P* = .27)
Within guidelines	1926	(77.3)			(71.6)	1.24 (*P* = .27)
Physical activity						
Meets guidelines	927	(30.6)			(58)	29.93 (*P* < .001)
Does not meet guidelines	2101	(69.4)			(42)	29.93 (*P* < .001)
Diet (‘5‐a‐day’)						
Yes	835	(27.6)			(29)	0.05 (*P* = .83)
No	2193	(72.4)			(71)	0.05 (*P* = .83)
Number of health behaviour guidelines breached ^g^			1.7	(0.83)		
1	828	(27.3)				
2	1304	(43.1)				
3	616	(20.3)				
4	109	(3.6)				
5	5	(0.2)				
No guidelines breached	166	(5.5)				

^a^ National data in relation to gender retrieved from:https://www.ons.gov.uk/peoplepopulationandcommunity/populationandmigration/populationestimates/datasets/populationestimatesforukenglandandwalesscotlandandnorthernireland

^b^ National data in relation to social grade retrieved from:http://www.nrs.co.uk/nrs‐print/lifestyle‐and‐classification‐data/social‐grade/

^c^ National data in relation to ethnicity retrieved from:https://www.ethnicity‐facts‐figures.service.gov.uk/

^d^ National data in relation to obesity, smoking, diet and physical activity calculated from pooled data from national health surveys for England, Scotland, Wales and Northern Ireland: https://www.gov.scot/publications/scottish‐health‐survey‐2017‐volume‐1‐main‐report/pages/98/; https://www.health‐ni.gov.uk/publications/health‐survey‐northern‐ireland‐first‐results‐201718; https://gov.wales/statistics‐and‐research/national‐survey/?tab=current&lang=en;https://digital.nhs.uk/data‐and‐information/publications/statistical/health‐survey‐for‐england/2017

^e^ Excludes n = 113 who were underweight and n = 190 who did not provide height and/or weight data. Total n = 2725

^f^ Defined according to participants drinking habits on a typical day, in line with government guidelines, where more than 8 units for men and more than 6 units for women (more than 4 units for men, and more than 3 units for women in Northern Ireland) constitutes increased risk. Excludes n = 536 who reported not consuming any alcohol. Total n = 2492.

^g^ According to health behaviours deemed to be high risk; scored out of five.

^h^ According to the National Readership Survey (http://www.nrs.co.uk/nrs‐print/lifestyle‐and‐classification‐data/social‐grade/); A; Higher managerial, administrative and professional; B; Intermediate managerial, administrative and professional; C1; Supervisory, clerical and junior managerial, administrative and professional; C2; Skilled manual workers; D; Semi‐skilled and unskilled manual workers; E; State pensioners, casual and lowest grade workers, unemployed with state benefits only.

### 3.2 Patients' health behaviours

Table 1 shows an overview of participants' BMI (BMI ≥ 25 compared to BMI < 25) and their health behaviours, including smoking status, fruit and vegetable consumption (in accordance with the ‘5‐a‐day’ recommendations,^21^ amount of physical activity (meeting national guidelines) and alcohol consumption (exceeding recommended units on heaviest drinking day). Participants' mean BMI was 27.50 (>25 is overweight according to NHS categories^22^). More than half of the sample (58.3%; n = 1767) was overweight or obese, 15.7% (n = 476) were smokers, 27.6% (n = 835) did not meet national guidelines for fruit and vegetable consumption (‘five‐a‐day’), 69.4% (n = 2101) did not meet physical activity guidelines and 22.7% (n = 566) of the sample reported excess alcohol consumption. Overall, 94.5% (n = 2862) of people who had recently attended a GP appointment breached at least one national health behaviour guideline, 67.2% (n = 2034) breached two or more and 24.1% (n = 730) breached three or more (shown in Table 1).

### 3.3 Patients' experiences of discussion about health behaviours during consultations

Table 2 shows an overview of patients' experiences of GP appointments. Participants reported a mean of nine appointments in the preceding 12 months. The mean EQ‐VAS score was 59.22, and specific reasons for the GP visit included to discuss a chronic condition (n = 1843; 60.9%), to discuss an acute condition (n = 326; 10.8%) or another reason (n = 587; 19.4%). The most commonly reported chronic conditions, as the purpose for the GP visit, were depression (n = 481; 15.9%), anxiety (n = 415; 13.7%), arthritis (n = 293; 9.7%), type 2 diabetes (n = 200; 6.6%) and asthma (n = 173; 5.7%).

**TABLE 2:** Patients' experiences of receiving health behaviour interventions during routine GP appointments

Variable	n	%	Mean (range, if applicable)	(SD)
Total GP visits (previous 12 months)			9.27 (1‐100)^a^	(12.80)
Number of times GP asked about health behaviours (all appointments in previous 12 months)^a^			5.41 (0‐50)	(9.11)
Current health (0‐100)^b^			59.22 (0‐100)	(22.74)
Reason for GP appointment				
Chronic condition^c^	1843	(60.9)		
Acute condition	326	(10.8)		
Other reason (eg one‐off visits)	587	(19.4)		
Most commonly reported chronic condition as purpose for visit^c^				
Depression	481	(15.9)		
Anxiety	415	(13.7)		
Arthritis	293	(9.7)		
Type 2 Diabetes	200	(6.6)		
Asthma	173	(5.7)		
Number of times GP asked about health behaviour (all appointments in previous 12 months; grouped)^d^				
Never	1578	(55.1)		
Once	941	(32.9)		
Twice	144	(5.0)		
More than twice	199	(7.0)		
Mean number of times GP asked about health behaviours (per appointment; calculated as number of times asked/number of appointments)^d^			0.47 (*less than once*)	(2.76)
Health behaviours discussed during last GP visit				
Yes	1732	(57.2)		
No	1296	(42.8)		
Specific health behaviours discussed during last GP visit				
Alcohol	2875	(94.9)		
Diet	846	(27.9)		
Physical activity	1070	(35.3)		
Smoking	1128	(37.3)		
Weight management (loss)	704	(23.2)		
Weight management (gain)	780	(25.8)		
Did your doctor (GP) refer you somewhere else for further advice or information about your health behaviour?				
Yes	424	(14)		
No	1254	(41.4)		
Don't know	54	(1.8)		
Did not answer	1296	(42.8)		

^a^ Of the total sample, 16 people (0.5% of the sample) reported over 90 visits in the previous 12 months, but we were unable to confirm the validity of these responses. For completeness, we have kept these data in the final analyses.

^b^ According to EQ‐VAS

^c^ According to the Functional Comorbidity Index, plus ‘other chronic conditions’ indicated by respondents.

^d^ According to participants who breached at least one national guideline in relation to health behaviour, according to alcohol, diet, physical activity, smoking and weight management (n = 2862).

Participants reported a mean of five occasions on which GPs asked about their health behaviours (across all appointments in the preceding 12 months); approximately half of all appointments (M = 0.47; SD = 2.76). Over half of the sample (57.2%; n = 1732) reported that health behaviour was discussed during their last GP appointment (57.2%; n = 1732). Where health behaviour was discussed in their last GP visit, a referral for further advice or information about health behaviour was reported by 14% (n = 424) of our sample. The most commonly reported behaviour discussed during the consultation was alcohol intake (n = 2875; 94.9% of the sample), followed by smoking (n = 1128; 37.3% of the sample) and physical activity (n = 1070; 35.3% of the sample). Despite the fact that the average patient was overweight, the least commonly discussed health issue was weight loss (n = 704; 23.2% of the sample).

### 3.4 Patients' experiences of discussing health behaviours during consultations in relation to current health behaviour status

Of the patients who had breached at least one national health behaviour guideline (94.5%; n = 2862), and would therefore benefit from behaviour change advice, 55.1% (n = 1578) reported never having had a conversation with their GP about health behaviours (Table 2). We therefore examined the extent to which GPs discussed the health behaviours that patients believed they needed to change (Table 3). Regardless of which health behaviour guideline was breached, alcohol intake was the mostly commonly discussed topic (range 90.8%‐95.2% across behaviours). For example, 208/476 (43.7%) of current smokers reported having a discussion with their GP about their smoking, but 90.8% of current smokers were asked about their alcohol consumption. For those patients not meeting ‘5‐a‐day’ recommendations (n = 835), 224 (26.8%) reported having a discussion with their GP about their diet. Amongst patients with a BMI of ≥25 (n = 1767), 388 (22.0%) patients reported weight loss discussions, 477 (27.0%) about diet and 626 (35.4%) about physical activity. Amongst patients not meeting physical activity guidelines (n = 2101), 743 (35.4%) patients reported physical activity discussions. For patients consuming alcohol to excess (n = 566), 532 (94.0%) patients reported having a discussion with their GP about their alcohol consumption.

**TABLE 3:** Health behaviour interventions received during last GP visit according to patients' current health behaviour status

Health behaviour discussed	Smoking				Diet (‘5‐a‐day’)				Weight				Physical activity				Alcohol intake			
	Non‐smokers (n = 2552)		Smokers (n = 476)		Yes (n = 2193)		No (n = 835)		BMI < 25 (n = 1261)		BMI ≥ 25 (n = 1767)		Meets guidelines (n = 927)		Does not meet guidelines (n = 2101)		Within guidelines (n = 2462)		Excessive consumption (n = 566)	
	n	%	n	%	n	%	n	%	n	%	n	%	n	%	n	%	n	%	n	%
Smoking	920	36.1	208	43.7	820	37.4	308	36.9	462	36.6	666	37.7	371	40.0	757	36.0	889	36.1	239	42.2
Diet	666	26.1	180	37.8	622	28.4	224	26.8	369	29.3	477	27.0	265	28.6	581	27.7	618	25.1	228	40.3
Weight loss	475	18.6	229	48.1	543	24.8	161	19.3	316	25.1	388	22.0	203	21.9	501	23.8	532	21.6	172	30.4
Physical activity	871	34.1	199	41.8	797	36.3	273	32.7	444	35.2	626	35.4	327	35.3	743	35.4	829	33.7	241	42.6
Alcohol intake	2443	95.7	432	90.8	2080	94.8	795	95.2	1200	95.2	1675	94.8	879	94.8	1996	95.0	2343	95.2	532	94.0

### 3.5 Patients' perceptions of receiving health behaviour change interventions

As shown in Table 4, behaviour change interventions were perceived favourably for all health behaviours. Participants perceived advice as either ‘very appropriate’ or ‘appropriate’ (range 81.5%‐88.8% across behaviours, regardless of whether people were breaching national guidelines for health behaviour). Intervention was perceived as either ‘very helpful’ or ‘helpful’ (range 77.7%‐87.3% across behaviours), regardless of whether breaching national guidelines for health behaviour. For those participants who reported receiving advice (n = 1732), participants reported low levels of concern (M = 3.57, SD = 2.67) about receiving advice about health behaviours from a GP. Concerning patients' expectations of receiving advice about health behaviours, the mean score corresponded to the scale mid‐point (M = 6.12, SD = 2.54). Thus, in general, patients perceive behaviour change intervention from their GP as appropriate and helpful.

**TABLE 4:** Perceptions of receiving behaviour change interventions during routine GP consultations according to patients breaching each national health behaviour guideline

	Smoking				Diet (‘5‐a‐day’)				Weight				Physical activity				Alcohol intake			
	Non‐smokers (n = 2552)		Smokers (n = 476)		Yes (n = 2193)		No (n = 835)		BMI < 25 (n = 1261)		BMI ≥ 25 (n = 1767)		Meets guidelines (n = 927)		Does not meet guidelines (n = 2101)		Within guidelines (n = 2462)		Excessive consumption (n = 566)	
	n	%	n	%	n	%	n	%	n	%	n	%	n	%	n	%	n	%	n	%
Helpfulness^a^																				
Not at all helpful	50	(2.0)	13	(2.7)	26	(1.2)	11	(1.3)	24	(1.9)	20	(1.1)	9	(1.0)	32	(1.5)	39	(1.6)	10	(1.8)
Not helpful	45	(1.8)	12	(2.5)	62	(2.8)	12	(1.4)	37	(2.9)	37	(2.1)	18	(1.9)	58	(2.8)	43	(1.7)	26	(4.6)
Neither helpful nor unhelpful	227	(8.9)	81	(17.0)	308	(14.0)	89	(10.7)	189	(15.0)	226	(12.8)	95	(10.2)	309	(14.7)	290	(11.8)	68	(12.0)
Helpful	971	(38.0)	197	(41.4)	1007	(45.9)	346	(41.4)	506	(40.1)	811	(45.9)	376	(40.6)	955	(45.5)	1037	(42.1)	232	(41.0)
Very helpful	1259	(49.3)	173	(36.3)	790	(36.0)	377	(45.1)	505	(40.0)	673	(38.1)	429	(46.3)	747	(35.6)	1053	(42.8)	230	(40.6)
Appropriateness^b^																				
Not at all appropriate	77	(3.0)	14	(2.9)	29	(1.3)	14	(1.7)	34	(2.7)	15	(0.8)	15	(1.6)	29	(1.4)	53	(2.2)	15	(2.7)
Not appropriate	32	(1.3)	13	(2.7)	49	(2.2)	11	(1.3)	25	(2.0)	33	(1.9)	14	(1.5)	47	(2.2)	39	(1.6)	29	(5.1)
Neither appropriate nor inappropriate	186	(7.3)	57	(12.0)	273	(12.4)	69	(8.3)	174	(13.8)	198	(11.2)	81	(8.7)	285	(13.6)	253	(10.3)	57	(10.1)
Appropriate	841	(33.0)	193	(40.5)	977	(44.6)	337	(40.4)	477	(37.8)	788	(44.6)	363	(39.2)	964	(45.9)	983	(39.9)	223	(39.4)
Very appropriate	1416	(55.5)	199	(41.8)	865	(39.4)	404	(48.4)	551	(43.7)	733	(41.5)	454	(49.0)	776	(36.9)	1134	(46.1)	242	(42.8)

^a^ Participants were asked: ‘In general, how helpful or not do you think it is for doctors (GPs) to ask people about the following?’

^b^ Participants were asked: ‘In general, how appropriate or not do you think it is for doctors (GPs) to ask people about the following?’

### 3.6 Patients' perceptions of receiving health behaviour change interventions (amongst patients who received interventions)

As shown in Table 5, among patients who actually received behaviour change interventions and who were breaching guidelines (smoking cessation, diet, weight loss, physical activity, and alcohol reduction), intervention was perceived as ‘very appropriate’ or ‘appropriate’ (range 79.3%‐87.1%) and ‘very helpful’ or ‘helpful’ (range 80.3%‐83.9%). These results are also comparable to the perceptions of patients who were within national guidelines for health behaviour, but were in any case asked to change their behaviour. Across all health behaviours, intervention was perceived as ‘very appropriate’ or ‘appropriate’ (range 77.6%‐88%) and ‘very helpful’ or ‘helpful’ (range 76.2%‐87.9%). Thus, even when GPs provided what might be considered unnecessary intervention (eg asking non‐smokers to quit), it seems there was little offence taken on the part of the patient.

**TABLE 5:** Perceptions of receiving behaviour change interventions during routine GP consultations according to patients breaching each national health behaviour guideline (and according to patients who received advice)

	Smokers who received smoking cessation advice (n = 208)		Non‐smokers who received smoking cessation advice (n = 920)		Patients not meeting ‘5‐a‐day’ who received dietary advice (n = 224)		Patients meeting ‘5‐a‐day’ who received dietary advice (n = 622)		Patients with a BMI < 25 who received weight loss advice (n = 388)		Patients with a BMI ≥ 25 who received weight loss advice (n = 316)		Patients not meeting physical activity guidelines, who received advice (n = 743)		Patients meeting physical activity guidelines, who received advice (n = 327)		Patients exceeding alcohol intake guidelines who received advice (n = 532)		Patients within alcohol intake guidelines who received advice (n = 2343)	
	n	%	n	%	n	%	n	%	n	%	n	%	n	%	n	%	n	%	n	%
Helpfulness^a^																				
Not at all helpful	5	(2.4)	21	(2.3)	2	(0.9)	5	(0.8)	3	(0.8)	5	(1.6)	8	(1.1)	3	(0.9)	9	(1.7)	37	(1.6)
Not helpful	4	(1.9)	17	(1.8)	5	(2.2)	23	(3.7)	4	(1.0)	15	(4.7)	22	(3.0)	12	(3.7)	24	(4.5)	36	(1.5)
Neither helpful nor unhelpful	32	(15.4)	73	(7.9)	29	(12.9)	90	(14.5)	63	(16.2)	55	(17.4)	92	(12.4)	36	(11.0)	64	(12.0)	268	(11.4)
Helpful	79	(38.0)	327	(35.5)	76	(33.9)	279	(44.9)	158	(40.7)	112	(35.4)	333	(44.8)	126	(38.5)	218	(41.0)	999	(42.6)
Very helpful	88	(42.3)	482	(52.4)	112	(50.0)	225	(36.2)	160	(41.2)	129	(40.8)	288	(38.8)	150	(45.9)	217	(40.8)	1003	(42.8)
Appropriateness^b^																				
Not at all appropriate	4	(1.9)	36	(3.9)	8	(3.6)	9	(1.4)	2	(0.5)	11	(3.5)	7	(0.9)	9	(2.8)	15	(2.8)	48	(2.0)
Not appropriate	5	(2.4)	16	(1.7)	3	(1.3)	23	(3.7)	9	(2.3)	11	(3.5)	23	(3.1)	9	(2.8)	27	(5.1)	35	(1.5)
Neither appropriate nor inappropriate	24	(11.5)	59	(6.4)	18	(8.0)	78	(12.5)	54	(13.9)	49	(15.5)	83	(11.2)	29	(8.9)	52	(9.8)	234	(10.0)
Appropriate	78	(37.5)	272	(29.6)	75	(33.5)	271	(43.6)	153	(39.4)	108	(34.2)	324	(43.6)	128	(39.1)	209	(39.3)	947	(40.4)
Very appropriate	97	(46.6)	537	(58.4)	120	(53.6)	241	(38.7)	170	(43.8)	137	(43.4)	306	(41.2)	152	(46.5)	229	(43.0)	1079	(46.1)

### 3.7 Factors associated with recall of receipt of behaviour change interventions

#### 3.7.1 Age, gender, ethnicity and social grade

Table 6 shows the results of a binary logistic regression analysis on which receipt of behaviour change intervention during patients' previous GP appointment was regressed on sociodemographic variables and perceptions of acceptability. Men compared with women (OR = 1.412, 95% CI 1.217, 1.639) and people of lower social grade compared with higher social grade (OR = 1.352, 95% CI 1.162, 1.572) were more likely to report behaviour change advice during their last GP appointment. However, people older than 64 compared with people younger than 25 (OR = 0.589, 95% CI 0.440, 0.787), and people of white ethnic background were less likely to report behaviour change advice during their last GP appointment.

**TABLE 6:** Factors associated with receipt of behaviour change intervention during last GP visit

Variables	Odds ratio (95% CI)	
	CI	*β*
Gender (men)	1.217, 1.639	1.412***
Age (years; ref: 18‐25)		
26‐34	0.742, 1.414	1.024
35‐44	0.710, 1.327	0.971
45‐54	0.809, 1.492	1.099
55‐64	0.651, 1.188	0.879
>64	0.440, 0.787	0.589***
Ethnicity (White)	0.330, 0.699	0.480***
Social grade (manual)	1.162, 1.572	1.352***
Presence of chronic long‐term condition^a^	1.287, 1.782	1.514***
Number of GP visits in previous year	1.010, 1.023	1.016***
Smoker (yes)	1.588, 2.419	1.960***
Alcohol (high risk)	1.336, 1.959	1.618***
High BMI (yes)	0.921, 1.247	1.072
Physical activity (high risk)	0.857, 1.196	1.012
Perceived appropriateness^b^	1.002, 1.048	1.025*
Perceived helpfulness^b^	1.003, 1.040	1.022*

^a^ According to the Functional Comorbidity Index.

^b^ Composite scores of perceptions of appropriateness and helpfulness were calculated across all health behaviours to create a single outcome (respective Cronbach's alpha scores were very good; 0.947 and 0.962, respectively.

* *P* < .05,

*** *P* < .001.

#### 3.7.2 Current health status and perceptions of acceptability

People who reported that their last GP visit was to discuss a chronic health condition, compared with those who did not (OR = 1.514, 95% CI 1.287, 1.782), were more likely to report receiving a behaviour change intervention during the consultation; total number of GP visits in the previous year was also associated with recalling receipt of behaviour change intervention (OR = 1.016, 95% CI 1.010, 1.023). People who were smokers compared with non‐smokers (OR = 1.960, 95% CI 1.588, 2.419) and people who reported exceeding alcohol guidelines compared with those who did not (OR = 1.618, 95% CI 1.336, 1.959) were more likely to report receiving a behaviour change intervention. People reporting higher levels of perceived appropriateness (OR = 1.025, 95% CI 1.002, 1.048) and perceived helpfulness (OR = 1.022, 95% CI 1.003, 1.040), compared with people who did not, were more likely to report receiving a behaviour change intervention at their last GP appointment. However, there were no differences in the likelihood of recalling receipt of interventions based on perceived appropriateness and helpfulness. Conversely, there were no differences in the likelihood of perceiving interventions as appropriate and helpful based on receipt of interventions.

## 4 DISCUSSION

### 4.1 Summary

This is the first study to (a) identify the characteristics of patients attending routine GP appointments, (b) assess patient need for behaviour change intervention according to national guidance, (c) examine patients' perceptions of acceptability behaviour change intervention during routine GP consultations and (d) identify factors associated with recall of receipt of behaviour change interventions. There were four important findings. First, our sample closely resembled the general population in terms of gender, ethnicity, social grade, BMI, smoking status, alcohol consumption and diet. However, there were four important differences which illustrate the characteristics of patients attending GP appointments; our sample contained fewer younger adults and more older adults, more people who were more physically inactive, more people in social grade A, and poorer health in general, compared to the general population. Second, of the patients who had breached at least one national health behaviour guideline (94.5% of the total sample), 55.1% reported never having had a conversation with their GP about health behaviours. Third, for all health behaviours regardless of whether patients received advice from their GP or not, patients perceived behaviour change advice as appropriate and helpful. Fourth, there are variations in the likelihood of receiving a behaviour change intervention according to sociodemographic factors.

### 4.2 Strengths and limitations

To our knowledge, this is the first attempt to examine patients' willingness to receive opportunistic behaviour change interventions during routine GP consultations and to understand the demographic profile of patients attending GP appointments. Findings demonstrate an unmet patient need to deliver behaviour change interventions as part of routine medical consultations. Our findings show that almost our entire sample (94.5%; n = 2862) breached at least one health behaviour guideline and may subsequently benefit from behaviour change advice. Future research should aim to build on these findings to develop effective screening tools and brief behaviour change interventions that will enable GPs and other health‐care professionals quickly to identify and deliver behaviour change interventions to the patients who would benefit most. One approach might be to look further in depth as to why it is that alcohol‐related interventions are more often deployed by GPs than interventions for other health behaviours.

There are limitations to this study. Participants were identified from a pre‐existing sample of the general public who were recruited and incentivized by YouGov to complete the questionnaire. The sample therefore may not be fully representative of all people who had recently attended a GP appointment. However, YouGov attempted to overcome this by seeking the widest possible variation in terms of demographic characteristics.

We were able to compare our sample with general population data; our sample closely resembled the general population in terms of gender, ethnicity and social grade (with the exception of ‘social grade A’). Additionally, our sample closely resembled national figures for BMI, smoking status, alcohol consumption and diet. Our sample included a higher proportion of people not meeting physical activity guidelines, a lower proportion of adults aged 18‐34, a higher proportion of adults aged 55‐64 and a higher proportion of people in social grade A, compared to national data. These differences may highlight important demographic factors of people attending GP appointments, compared with the population more generally. We were unable to identify data on how frequently people visit their GP, but our sample is likely to be reasonably representative considering repeat visits among people with long‐term health conditions (60.9% of our sample), and that our sample reported poorer health in general (*m* = 59.22) than the UK population at large (*m* = 82.5).^23^ A further limitation is that GPs' expectations regarding patient perceptions of receiving health behaviour interventions is just one potential barrier to widespread delivery of health behaviour change interventions in general practice. Findings must therefore be considered in light of the time constraints and priorities of routine GP consultations. Finally, the self‐report nature of the present study relied on subjective measures of behaviour, that may be influenced by other aspects not captured within this study, such as the time since the appointment (which may influence recall). The gap between GPs' and patients' perceptions is likely to be narrower than that of GPs perception and patients' actual health behaviours.^24^ Future research and practice would therefore benefit from employing objective, validated measures that could, for example, be administered during routine GP appointments. This would allow a more accurate representation of patients' *actual* health behaviours, as opposed to *perceived* health behaviours.

### 4.3 Comparison with existing literature

Our findings suggest there is a considerable unmet need to address behaviour change during GP consultations, which is a cause for concern. A recent national survey of health‐care professionals reported that GPs perceived that 44% of patients seen in a typical week would benefit from a behaviour change intervention.^9^ However, our findings suggest there is a considerable mismatch between health‐care professionals' perceptions of patient benefit for behaviour change interventions and patients' perceived need; almost our entire sample (94.5%) breached at least one national guideline and would consequently benefit from an intervention (more than double the proportion estimated by GPs [44%], but comparable to the proportion [87%] reported in the National Health Survey for England^3^). This supports public health policy, which compels health‐care professionals to offer opportunistic behaviour change interventions during routine medical consultations.^4^ Additional concerns are the findings in relation to behaviour change discussion during consultations. Of the patients breaching at least one national guideline for health behaviour, 55.1% of patients reported never having had a conversation with their GP about health behaviours in the preceding 12 months. This is particularly important given that 64.8% of our sample had a BMI indicative of high risk, 69.4% of patients did not meet physical activity guidelines, and 72.4% of patients did not meet national guidelines for dietary intake.

### 4.4 Implications for practice

Behaviour change interventions delivered by GPs have been shown to be effective in changing patients' behaviour.^19^, ^25^ Whilst there is ambivalence amongst GPs about delivering behaviour change interventions in routine consultations,^5^, ^8^ we found evidence that interventions are being delivered during some consultations, even if the proportion is low. Our findings show that smokers (compared with non‐smokers) and people exceeding alcohol guidelines (compared with those who are not) were more likely to report receiving a behaviour change intervention during their last GP visit. However, the skills required to deliver behaviour change interventions opportunistically may not be a part of health‐care professional core training or practice^5^, ^26^; education and training to deliver interventions must therefore take priority.^27^

Specific reasons contributing to the likelihood of GPs delivering interventions may include a fear of offending the patient,^28^ beliefs that patients lack the motivation for behaviour change^5^ and perceptions that patients do not want or need behaviour change interventions.^10^, ^11^ Our study suggests that patients (a) are receptive and would welcome a discussion about behaviour change, and (b) where behaviour change was discussed with patients, this was rated favourably by patients, even in cases of less personally relevant health behaviours. Further, GPs underestimate the proportion of patients that would benefit from a behaviour change intervention, and there are considerable differences in patient need compared to the proportion of patients breaching health behaviour guidelines observed in the current study.

## 5 CONCLUSIONS

Behaviour change interventions delivered by GPs during routine medical consultations enable interventions to have maximum reach and can be used effectively when incorporated into time‐restricted consultations.^19^ Our findings suggest that GPs underestimate the proportion of patients that would benefit from behaviour change interventions, which patients perceive as appropriate and helpful. GPs could adopt a more proactive approach to behaviour change in patient consultations with the broad approval of patients.

## ETHICS APPROVAL AND CONSENT TO PARTICIPATE

Ethical approval for the study was obtained from a university ethics committee (ref. 2017‐0739‐1780).

## COMPETING INTERESTS

The authors declare that they have no competing interests.

## AUTHORS' CONTRIBUTION

CK and CJA conceived the study. CK was responsible for data curation and leading data analysis. CK, TE, JG, RC, and CJA contributed to design and methodology. CK and CJA led the first draft of the manuscript, and all study authors contributed to later drafts and agreed on the final version.

## Data Availability

The data that support the findings of this study are available on request from the corresponding author. The data are not publicly available due to privacy or ethical restrictions.
